# Electrical reconnection after pulsed field ablation with a circular over-the-wire catheter in atrial fibrillation

**DOI:** 10.1093/europace/euaf216

**Published:** 2025-09-12

**Authors:** Marisa van der Graaf, Jason G Andrade, Seth Kibel, Matthew T Bennett, Richard G Bennett, Bob G S Abeln, Jippe C Balt, Maurits C E F Wijffels, Max Liebregts, Vincent F van Dijk, Lucas V A Boersma

**Affiliations:** Department of Cardiology, St. Antonius Hospital, Koekoekslaan 1, Nieuwegein 3435 CM, The Netherlands; Department of Cardiology, Amsterdam UMC, Amsterdam, The Netherlands; Electrophysiology Service, Department of Cardiology, Montreal Heart Institute, Université de Montréal, Montreal, Quebec, Canada; Dilawri Cardiovascular Institute, Vancouver General Hospital, Vancouver, BC, Canada; Dilawri Cardiovascular Institute, Vancouver General Hospital, Vancouver, BC, Canada; Dilawri Cardiovascular Institute, Vancouver General Hospital, Vancouver, BC, Canada; Dilawri Cardiovascular Institute, Vancouver General Hospital, Vancouver, BC, Canada; Department of Cardiology, St. Antonius Hospital, Koekoekslaan 1, Nieuwegein 3435 CM, The Netherlands; Department of Cardiology, Amsterdam UMC, Amsterdam, The Netherlands; Department of Cardiology, St. Antonius Hospital, Koekoekslaan 1, Nieuwegein 3435 CM, The Netherlands; Department of Cardiology, St. Antonius Hospital, Koekoekslaan 1, Nieuwegein 3435 CM, The Netherlands; Department of Cardiology, St. Antonius Hospital, Koekoekslaan 1, Nieuwegein 3435 CM, The Netherlands; Department of Cardiology, St. Antonius Hospital, Koekoekslaan 1, Nieuwegein 3435 CM, The Netherlands; Department of Cardiology, St. Antonius Hospital, Koekoekslaan 1, Nieuwegein 3435 CM, The Netherlands; Department of Cardiology, Amsterdam UMC, Amsterdam, The Netherlands

**Keywords:** Atrial fibrillation, Pulmonary vein isolation, Pulsed field ablation, Recurrence procedure

## Introduction

Pulmonary vein isolation (PVI) is the cornerstone of catheter ablation treatment for patients with atrial fibrillation (AF). In recent years, PVI using pulsed field ablation (PFA) has gained widespread adoption as it mitigates risks associated with traditional thermal ablation methods.^1–3^ PFA lesions are created by the application of multiple short, high voltage electrical pulses to the myocardium, which selectively induce apoptosis of myocardial cells.^4^ Long-term outcomes on durability of PVI remain limited.^5^ This multicentre study provides an overview of repeat ablation procedural outcomes in patients with arrhythmia recurrence after a prior ablation procedure with the circular PulseSelect™ (Medtronic, Minneapolis, USA) PFA catheter.

## Methods

This is an international, multicentre, observational registry study involving two centres (St. Antonius Hospital, Nieuwegein, The Netherlands and Vancouver General Hospital, Vancouver, Canada) that use the commercially available PulseSelect™ catheter. Patients eligible for a repeat procedure after a prior PVI ablation with this catheter were included in this analysis. At each centre, the local ethics committee approved the collection of the required data, and the study was performed in accordance with the Declaration of Helsinki.

The PFA system uses a circular over-the-wire catheter, equipped with a nine-gold-plated electrode array and generates energy applications of biphasic, bipolar pulse trains at 1.5 kV. The initial PFA procedures were conducted according to the instructions for use and included four ostial and four antral applications for each pulmonary vein (PV). Additional applications could be delivered at operator discretion. No extrapulmonary vein structures were targeted.

Patients were considered eligible for a repeat ablation procedure in case of symptomatic recurrent atrial arrhythmias after a 2-month blanking period, as detected according to the local follow-up protocol (i.e. ECG, Holter, or photoplethysmography-based monitoring). All repeat procedures were performed with the use of a 3D mapping system (Carto 3, Biosense Webster Inc., Diamond Bar, CA, USA, or Ensite X, Abbott, Abbott Park, IL, USA) to evaluate PV electrical reconnection and guide re-isolation.

The primary endpoint was the number of patients with PV reconnection. Secondary endpoints included procedural characteristics, the specific location of gaps in isolation, and major adverse events.

Data analysis was performed using R, version 4.4.0 (R Foundation, Vienna, Austria). Outcomes are presented as mean ± SD or median [IQR] for continuous variables, and frequencies with percentages for categorical variables.

## Results

A total of 27/285 (9.5%) patients who underwent a repeat ablation before 28 May 2025, after a primary procedure between 29 January 2024 and 6 December 2024, were included in this analysis. The indication was recurrent AF in 17 (63%) and atrial flutter in 10 patients (37%). The cohort included 15 males (56%), the mean age was 65.0 ± 9.0 years and 14 (52%) had persistent AF prior to the initial procedure. The mean left atrial volume index was 39.9 ± 13.1 mL/m^2^, the mean left ventricular ejection fraction 53.0 ± 14.6% and two patients had a left common pulmonary vein. At baseline, PVI was achieved with a median total number of 34.0 [32.0; 37.0] applications.

**Figure 1:**
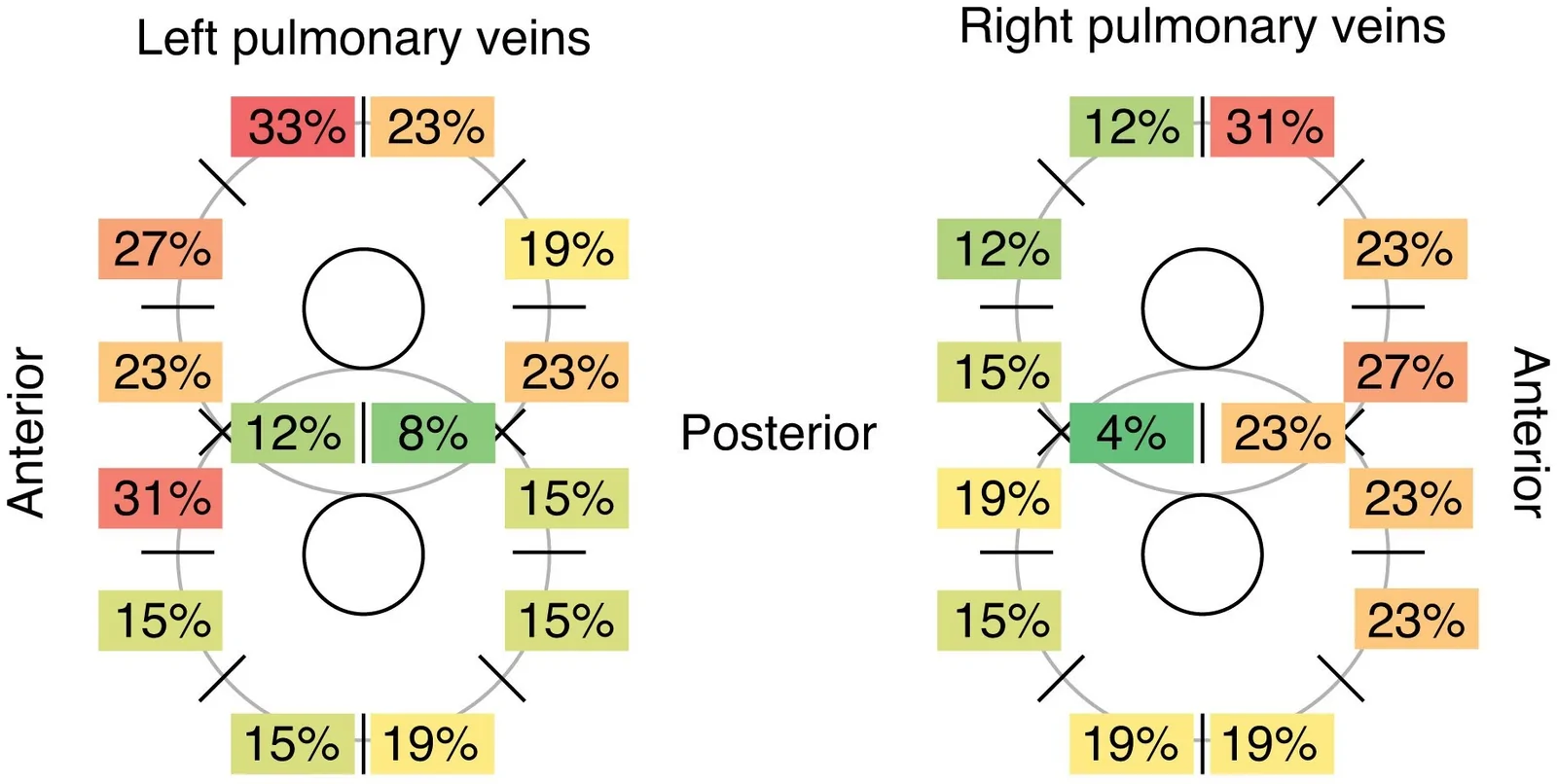
Segment based pulmonary vein gap assessment. Per segment, the percentage indicates the proportion of patients with reconnection of this segment.

The median time from the initial procedure to the repeat procedure was 172 [136; 212] days. In seven patients [25.9%, centre 1: 1/19 (5.3%), centre 2: 6/8 (75%)] all PVs were durably isolated, while in the remainder of patients at least one pulmonary vein showed reconnection: one PV in five patients (18.5%), two PVs in five patients (18.5%), three PVs in six patients (22.2%) and four PVs in four patients (14.8%). Gaps in PV isolation were most frequently observed at the anterior and superior side of the left PVs, and the anterior aspects of right PVs (*Figure 1*).

While baseline procedural characteristics were not significantly different between the centres, there was a trend towards a higher number of applications in centre 2 (36 [32; 41] vs. 34 [32; 35], *P* = 0.236), especially targeted to the carina and left atrial appendage (LAA) ridge.

No major adverse events occurred during and after the repeat ablation procedures.

## Discussion

We present an overview of repeat ablation outcomes in patients with symptomatic atrial arrhythmia recurrences after treatment with the PulseSelect PFA ablation catheter.

Recent articles on PulseSelect PFA procedures are mainly focusing on procedural characteristics, safety outcomes, and short-term efficacy outcomes.^6,7^ However, limited data are available regarding long-term efficacy outcomes including durability of PVI. Our results showed that reconnection of at least one PV was present in 74.1% of patients that underwent a repeat ablation procedure for recurrent atrial arrhythmias after an initial PulseSelect procedure. These numbers seem to be comparable to those with thermal ablation modalities and other contemporary PFA systems.^8–10^ The use of pre-procedural CT in centre 2, to guide catheter placement and lesion delivery during the primary procedure, may partially explain the lower rate of patients with at least one reconnected PV observed in this centre, as differences between centres could not be fully accounted for by procedural characteristics alone. Most gaps in isolation were identified at the anterior LSPV and anterosuperior LIPV, possibly due to suboptimal PFA catheter positioning near the LAA. Furthermore, gaps were also common at the anterior side of the right pulmonary veins, which may be explained by differential catheter contact in these regions. These findings underscore the importance of adequate contact for effective lesion formation, which can for example be facilitated by contact force sensing or additional applications.

## Limitations

For this study, we retrospectively collected data of patients who underwent a repeat procedure. We did not perform a standardized repeat procedure in all patients after PulseSelect PVI regardless of arrhythmia recurrence, which may not reflect the true PV reconnection rate. Secondly, since the PulseSelect PFA system is only commercially available since January 2024, the follow-up duration and the number of repeat ablation procedures are still limited. There may be more patients with late AF recurrence that may require repeat ablation after a longer follow-up time.

## Conclusion

This is the first study reporting repeat ablation outcomes after pulmonary vein isolation using the circular PulseSelect PFA system. Our findings highlight specific regions prone to reconnection, which may help operators optimize applications and improve long-term durability.

## Data Availability

The data underlying this article will be shared on reasonable request to the corresponding author.
