# Correction to: Electrical reconnection after pulsed field ablation with a circular over-the-wire catheter in atrial fibrillation

**DOI:** 10.1093/europace/euag281

**Published:** 2026-10-05

This is a correction to: Marisa van der Graaf, Jason G Andrade, Seth Kibel, Matthew T Bennett, Richard G Bennett, Bob G S Abeln, Jippe C Balt, Maurits C E F Wijffels, Max Liebregts, Vincent F van Dijk, Lucas V A Boersma, Electrical reconnection after pulsed field ablation with a circular over-the-wire catheter in atrial fibrillation, *EP Europace*, Volume 27, Issue 9, September 2025, euaf216, https://doi.org/10.1093/europace/euaf216

In the originally published version of this manuscript, the anterior and posterior gap locations in the right pulmonary veins (PVs) were inadvertently transposed in Figure 1.

Figure 1 has been corrected to read:

**Figure:**
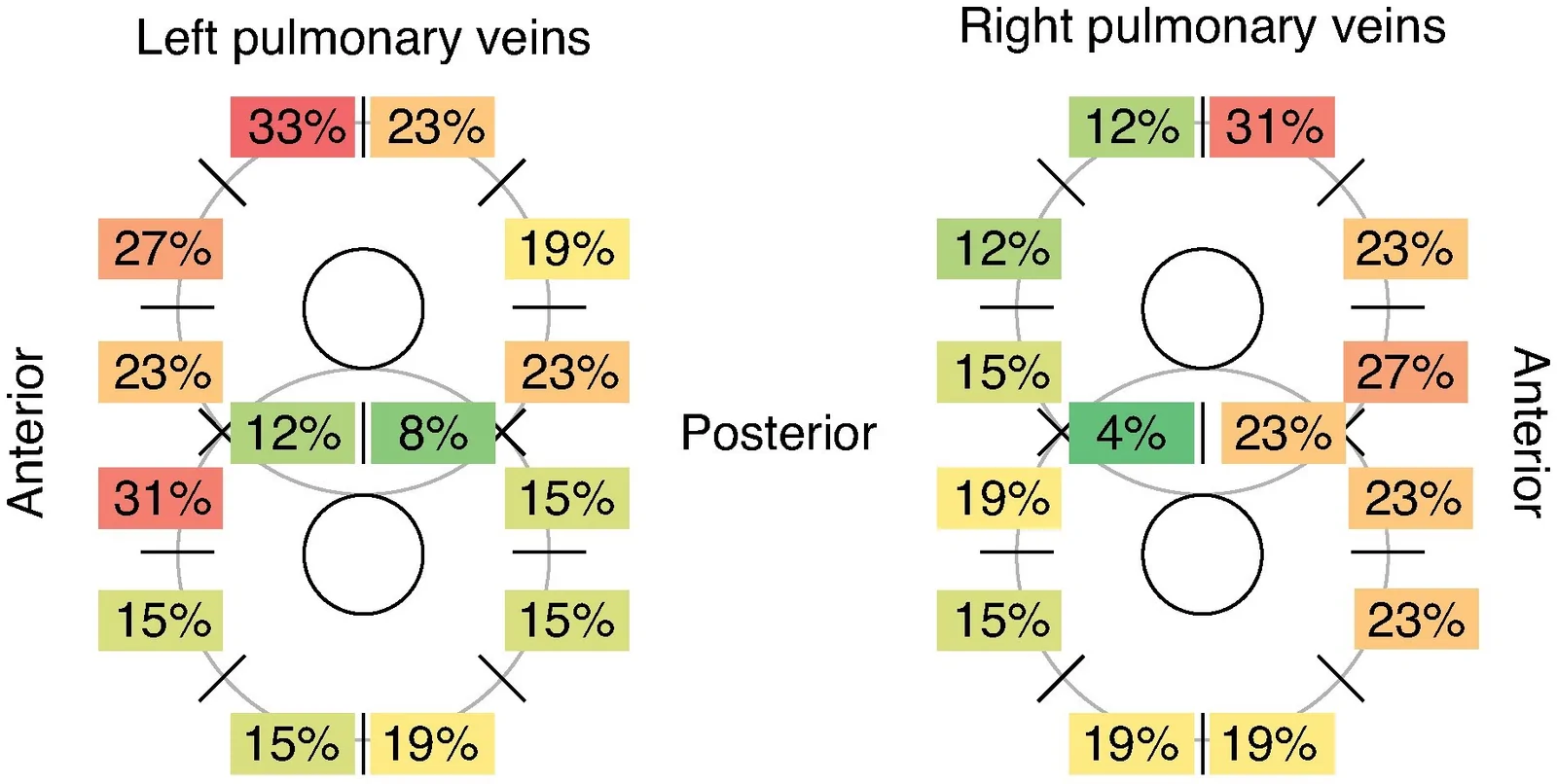


Instead of:

**Figure:**
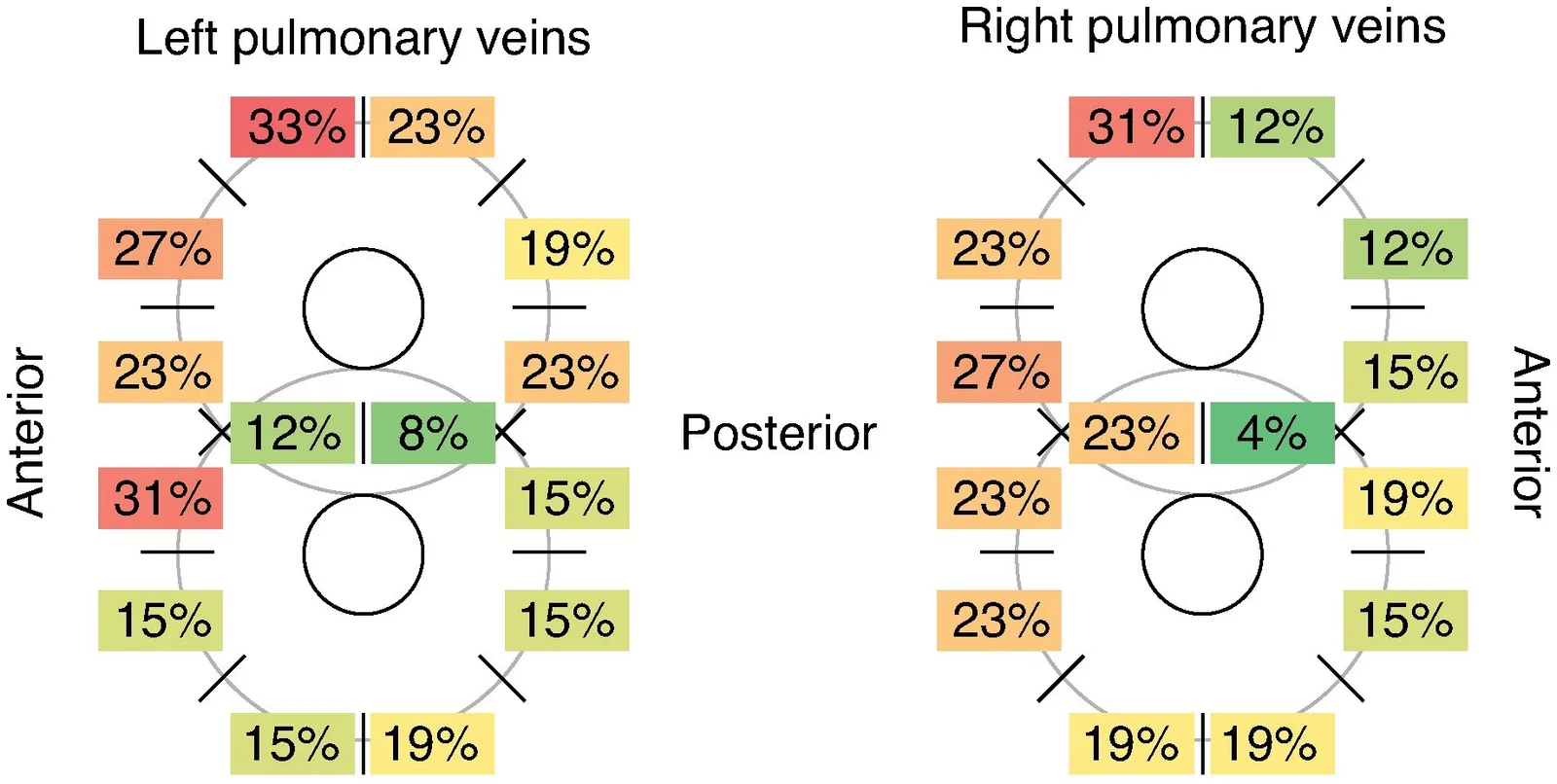


The corresponding sentence in the Results section has been corrected to read:

“Gaps in PV isolation were most frequently observed at the anterior and superior side of the left PVs, and the anterior aspects of right PVs (*Figure 1*)”

Instead of:

“Gaps in PV isolation were most frequently observed at the anterior and superior side of the left PVs, and the posterior aspects of right PVs (*Figure 1*)”.

The corresponding sentence in the Discussion section has been corrected to read:

“Furthermore, gaps were also common at the anterior side of the right pulmonary veins, which may be explained by differential catheter contact in these regions”

Instead of:

“Furthermore, gaps were also common at the posterior side of the right pulmonary veins, which may be explained by differential catheter contact in these regions”.

